# Genomic variants in the *FTO* gene are associated with sporadic amyotrophic lateral sclerosis in Greek patients

**DOI:** 10.1186/s40246-017-0126-2

**Published:** 2017-12-08

**Authors:** Konstantinos Mitropoulos, Eleni Merkouri Papadima, Georgia Xiromerisiou, Angeliki Balasopoulou, Kyriaki Charalampidou, Vasiliki Galani, Krystallia-Vassiliki Zafeiri, Efthymios Dardiotis, Styliani Ralli, Georgia Deretzi, Anne John, Kyriaki Kydonopoulou, Elpida Papadopoulou, Alba di Pardo, Fulya Akcimen, Annalisa Loizedda, Valerija Dobričić, Ivana Novaković, Vladimir S. Kostić, Clint Mizzi, Brock A. Peters, Nazli Basak, Sandro Orrù, Evangelos Kiskinis, David N. Cooper, Spyridon Gerou, Radoje Drmanac, Marina Bartsakoulia, Evangelia-Eirini Tsermpini, Georgios M. Hadjigeorgiou, Bassam R. Ali, Theodora Katsila, George P. Patrinos

**Affiliations:** 1 National and Kapodistrian University of Athens School of Medicine, Athens, Greece; 2 Department of Pharmacy, University of Patras School of Health Sciences, Campus, Rion, GR-26504 Patras, Greece; 3 School of Medicine, University of Thessaly, Larisa, Greece; 4 Papageorgiou hospital, Thessaloniki, Greece; 5 Department of Pathology, College of Medicine and Health Sciences, United Arab Emirates University, Al-Ain, UAE; 6 ANALYSI Diagnostic Laboratories S.A, Thessaloniki, Greece; 7 Departments of Neurology and Physiology, Northwestern University Feinberg School of Medicine, Chicago, IL USA; 8 Suna and Inan Kirac Foundation, NDAL, Bogazici University, Istanbul, Turkey; 9 Department of Medical Sciences and Public Health, University of Cagliari, Cagliari, Italy; 10 CNR IRGB, Cagliari, Italy; 11 Institute of Neurology CCS, School of Medicine, University of Belgrade, Belgrade, Serbia; 12 Faculty of Medicine, Institute of Human Genetics, University of Belgrade, Belgrade, Serbia; 13 Complete Genomics Inc., Mountain View, CA USA; 14 BGI Shenzhen, Shenzhen, People’s Republic of China; 15 Institute of Medical Genetics, Cardiff University, Cardiff, UK

**Keywords:** Sporadic amyotrophic lateral sclerosis, *FTO* gene, Genomic variants, Whole-genome sequencing, Founder population

## Abstract

**Background:**

Amyotrophic lateral sclerosis (ALS) is a devastating disease whose complex pathology has been associated with a strong genetic component in the context of both familial and sporadic disease. Herein, we adopted a next-generation sequencing approach to Greek patients suffering from sporadic ALS (together with their healthy counterparts) in order to explore further the genetic basis of sporadic ALS (sALS).

**Results:**

Whole-genome sequencing analysis of Greek sALS patients revealed a positive association between *FTO* and *TBC1D1* gene variants and sALS. Further, linkage disequilibrium analyses were suggestive of a specific disease-associated haplotype for *FTO* gene variants. Genotyping for these variants was performed in Greek, Sardinian, and Turkish sALS patients. A lack of association between *FTO* and *TBC1D1* variants and sALS in patients of Sardinian and Turkish descent may suggest a founder effect in the Greek population. *FTO* was found to be highly expressed in motor neurons, while in silico analyses predicted an impact on *FTO* and *TBC1D1* mRNA splicing for the genomic variants in question.

**Conclusions:**

To our knowledge, this is the first study to present a possible association between *FTO* gene variants and the genetic etiology of sALS. In addition, the next-generation sequencing-based genomics approach coupled with the two-step validation strategy described herein has the potential to be applied to other types of human complex genetic disorders in order to identify variants of clinical significance.

**Electronic supplementary material:**

The online version of this article (10.1186/s40246-017-0126-2) contains supplementary material, which is available to authorized users.

## Introduction

Amyotrophic lateral sclerosis (ALS), also known as Lou Gehrig’s disease, is a neurodegenerative disorder that affects the upper and lower motor neurons in the motor cortex, brain stem, and spinal cord [20]. ALS was first described in the mid-1800s as a rapidly progressing motor neuron disease, with phenotypes of varying severity. Affected patients present muscle weakness, atrophy, and a general progressive paralysis. Eventually, most develop respiratory failure which leads to death on average 3 to 5 years after the onset of symptoms [45]. As yet, there is no effective treatment or pre-symptomatic diagnostic test for ALS [11].

Approximately 90–95% of ALS patients suffer from a sporadic form of ALS (sALS), which has both an environmental etiology and a likely strong genetic component [23]. The remaining 5–10% of ALS patients exhibit a clear genetic etiology for the disease, also known as familial ALS (fALS). Genomic variants in the *SOD1* gene have been shown to lead to fALS, with an autosomal dominant mode of inheritance. To date, more than 170 different *SOD1* genomic variants have been reported, affecting ~ 13% of fALS and ~ 1% of sALS patients [40]. Furthermore, variants in other genomic loci, such as *FUS* [27], *TARDBP* [25], and more recently *C9orf72* [36], have also been implicated in fALS. In the case of *C9orf72*, a non-coding GGGGCC expansion appears to have a very strong association with both fALS and sALS, with an overall prevalence of 40 and 5–20% in fALS and sALS patients, respectively [39].

The etiology of sALS is quite complex. A variety of different factors, including age, overall lifestyle, occupation, and/or exposure to xenobiotics, appear to influence the risk of developing sALS, but no specific chemical or toxin has been identified as a direct cause of ALS [44]. In addition to environmental factors, sALS also has a substantial genetic component. Genome-wide association studies have so far identified over 100 genomic loci that could yet prove to be linked to ALS disease predisposition with low-to-medium penetrance, supporting the notion of pronounced heritability for ALS [reviewed in [28]]. From these genomic loci, *SQSTM1* [19], *UBQLN2* [14], *VCP* [12], *ANG* [29], *PRPH* [38], *DCTN1*, *TAF15*, and *SETX* [10] appear to be more frequently mutated in sALS than in fALS, with an overall prevalence of between 4 and < 1%; these findings have been confirmed in several studies/cohorts as well as in functional and animal studies. Furthermore, the co-occurrence of genomic variants in addition to the presence of de novo pathogenic variants in sALS brings the estimate of overall heritability for ALS to as high as 60% [3, 4]. The identification of the entire lexicon of underlying genetic defect(s) in both fALS and sALS patients and their families would greatly improve disease diagnosis and potentially disease management.

There is little knowledge of the genetic basis of ALS in fALS and sALS patients of Greek origin. Although the incidence of ALS in Greek patients is comparable to other European populations (~ 1–2 per 100,000), only a single study has so far been performed, highlighting a rather high prevalence of the *C9orf72* GGGGCC repeat expansion in Greek ALS patients [35]. Here, we report a novel genomic locus, *FTO*, that appears to be associated with sALS. Genomic data comparison with sALS patients of Greek, Sardinian, and Turkish descent strongly suggest that this association may represent a founder effect that is specific to the Greek population.

## Materials and methods

### Case selection

**Fig. 1:**
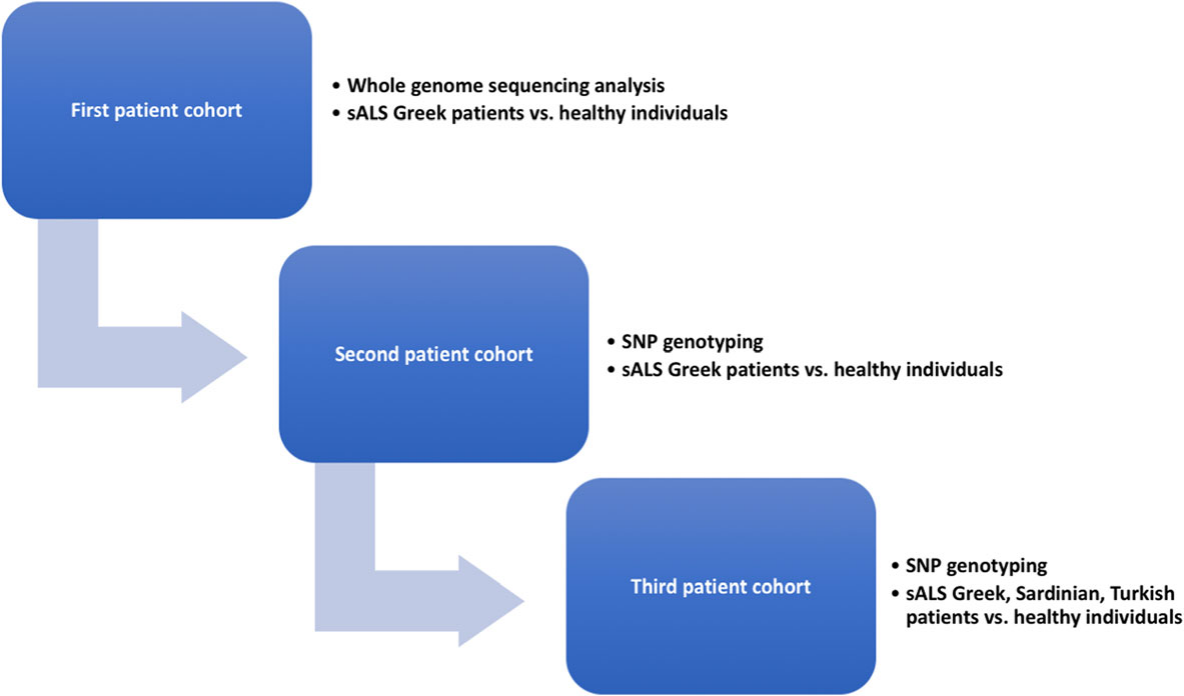
Our three-step strategy towards data reliability. Our three-step strategy involved three patient cohorts: (i) whole-genome sequencing analysis in sALS Greek patients (*n* = 6) and their healthy counterparts (*n* = 5), (ii) SNP genotyping (selected variants of interest) in sALS Greek patients (*n* = 28) and their healthy counterparts (*n* = 50), and (iii) SNP genotyping (selected variants of interest) in sALS patients of Greek (*n* = 114), Sardinian (*n* = 114), and Turkish (*n* = 148) origin and their healthy counterparts (*n* = 39, 87, 74, respectively). The first patient cohort served as the discovery dataset, while the second and third patient cohorts were the training datasets

Adopting a three-step strategy to determining data reliability, case selection criteria spanned all three patient cohorts studied herein (Fig. 1): (i) whole-genome sequencing analysis in sALS Greek patients (*n* = 6) and their healthy counterparts (*n* = 5), (ii) SNP genotyping (selected variants of interest) in sALS Greek patients (*n* = 28) and their healthy counterparts (*n* = 50), and (iii) SNP genotyping (selected variants of interest) in sALS patients of Greek (*n* = 114), Sardinian (*n* = 114), and Turkish (*n* = 148) origin and their healthy counterparts (*n* = 39, *n* = 87, *n* = 74, respectively). Patients were independently evaluated by at least two senior neurologists and diagnosed on the basis of revised El Escorial criteria [9]. Patients with definite, probable, probable laboratory supported, or possible ALS were included in the study. Healthy individuals were recruited after excluding the occurrence of common neurological disorders.

### Whole-genome sequencing and bioinformatics analyses

Genomic DNA isolation was performed either from peripheral blood using the QIAamp Blood Midi Kit (Qiagen GmbH, Hilden, Germany) or by means of the phenol-chloroform extraction method as previously described with minor modifications or saliva as previously described [1]. Whole-genome sequencing was performed using Complete Genomics’ (CA, USA) DNA nanoarray platform [18]. DNA sequencing coverage was ×110. Only high-quality call variants were included in the downstream analysis (> 93%). Genomes were aligned with the hg19 reference genome. Next-generation sequencing data (Complete Genomics Inc., CA) were analyzed using Ingenuity Variant Analysis version 3.1.2 (Ingenuity® Systems, www.ingenuity.com). This is well-established software that identifies associations between phenotypes, defined by the user in terms of the classification of the tested individuals, and variants in the sequenced genome. Upon classification by phenotype (sALS vs. healthy individuals), a number of variants were listed; the output was filtered into smaller variant lists of (i) those found only in sALS patients (*n* = 174 single nucleotide polymorphisms (SNPs)), (ii) those not previously annotated or annotated only once, (iii) those with matching Human Gene Mutation Database (HGMD) entries, and (iv) whole-exome findings. All variants were filtered according to the analysis required, using custom scripts and Complete Genomics Analysis Tools (CGA™ Tools).

### Linkage disequilibrium and in silico analyses

Pair-wise linkage disequilibrium (LD) calculations were based on phase genotype data (SNAP v2.2) [24], utilizing the HapMap Phase II + III (release 28) [6] and the 1000 Genomes Project (http://www.1000genomes.org) dataset for Caucasians (CEU). Findings were visualized on HaploView 4.2 and by using the LDmatrix module on the LDlink web tool [30]. Τo further investigate the role of the selected *FTO* and *TBC1D1* variants, we explored their effect on splicing motifs (including the “accept” and “donor” splice sites), the branch point, and auxiliary sequences that enhance (exonic splicing enhancers, ESE) or repress (exonic splicing silencers, ESS) splicing. To this end, in silico prediction was performed, using Human Splicing Finder (http://www.umd.be/HSF3/). This is a system that has rapidly become an international standard, as it combines 12 different algorithms [15]. Intronic variants, if “significant,” are often located within gene regions that are characterized by a reduced level of genetic variation. Conserved elements were therefore explored using Variant Effect Predictor [34].

### Downstream molecular genetic analysis

The first patient cohort served as our discovery dataset, while the second and third patient cohorts constituted our training datasets (Fig. 1). For this process, selected variants were subsequently validated in sALS Greek patients (*n* = 28) and their healthy counterparts (*n* = 50) (Fig. 1, Tables 1 and 2 and Additional file 1). PCR amplification was carried out according to the KAPA2G Fast HotStart protocol (KAPABIOSYSTEMS, MA, USA); detailed information regarding SNP amplification conditions is available upon request. For *FTO* rs2892469 (C>T) and rs1861869 (G>C), a PCR-based conventional Sanger resequencing approach was employed. Capillary electrophoresis was performed on an ABI Prism 3130xl DNA Analyzer (Applied Biosystems). For *FTO* rs17217144 (C>T) and rs7186521 (A>G) as well as *TBC1D1* rs6850200 (C>A), allele-specific PCR assays were developed (two alternative reverse primers hybridizing exclusively either to the wild-type or the mutant allele). Sanger sequencing was also employed to validate the allele-specific PCR method.

**Table 1:** Selected *FTO* and *TBC1D1* gene variants of prime interest

Gene	SNPs	Alleles	MAF-all	MAF-EUR	Variant type
*FTO*	rs2892469	C>T	0.31 (T)	0.49 (T)	Intronic
	rs1861869	C>G	0.38 (G)	0.54 (G)	
	rs17217144	C>T	0.31 (T)	0.49 (T)	
	rs7186521	A>G	0.37 (G)	0.49 (G)	
*TBC1D1*	rs6850200	C>A	0.41 (A)	0.37 (A)

**Table 2:** Genotyping data of sALS patients and their healthy counterparts

Population groups		Genotype frequency (%)																			
		*FTO* rs2892469				*FTO* rs1861869				*FTO* rs17217144				*FTO* rs7186521				*TBC1D1* rs6850200			
		CC	TT	CT	*p* value	CC	GG	CG	*p* value	CC	TT	CT	*p* value	AA	GG	AG	*p* value	AA	CC	CA	*p* value
Second patient cohort	Greek healthy individuals	26	36	38	0.005	21	41	38	0.003	0	0	100	PA = 1.55e^−12^ PB = 7.78e^−13^	0	0	100	1.0	18	45	38	0.017
	Greek sALS patients	17	22	61		17	22	61		22	13	65		0	0	100		9	35	57	
Third patient cohort	Greek healthy individuals	26	36	38	0.033	21	41	38	PA = 0.072 PB = 0.071	6	2	92	0.002	0	0	100	5.13e^*−39*^	14	43	43	0.723
	Greek sALS patients	21	23	56		17	29	54		22	2	76		18	65	18		11	42	48	
	Sardinian healthy individuals	38	25	38	0.63	36	31	33	0.61	24	36	40	0.67	35	22	42	0.64	20	41	39	0.53
	Sardinian sALS patients	31	28	41		29	34	37		27	30	43		29	25	46		15	39	46	
	Turkish healthy individuals	26	26	49	0.62	0	100	0	0.49	26	26	49	0.62	26	26	49	0.09	18	45	38	0.57
	Turkish sALS patients	24	21	55		0	99	1		26	20	54		21	26	53		13	44	43	

Sizing PCR across the *C9orf72* hexanucleotide repeats was performed using previously published primers [13] using repeat-primed PCR [32, 39]. Fragment length analysis was performed with the ABI 3500 genetic analyzer (Applied Biosystems, Foster City, Calif., USA) and analyzed using ABI GeneMapper v4.1 (Applied Biosystems).

### qRT-PCR expression analysis for the *FTO* and *TBC1D1* genes

The relative expression levels of *FTO* and *TBC1D1* were determined using human-specific primers in a range of various human cell types; fibroblasts, embryonic stem cells (ESCs), astrocytes, neural PAX6+ progenitors, stem cell-derived purified motor neurons at three different stages of development (*Hb9*::GFP on days 0, 16, and 30), and stem cell-derived cortical neurons (NKX21 on days 45 and 50). Human brain and spinal cord RNA served as controls. Cell pellets were resuspended in Trizol Reagent (Life Technologies), and RNA was isolated following the manufacturer’s protocol. 0.5 to 1 μg of RNA was treated with DNase I (Invitrogen) and subsequently used for the generation of cDNA using iSCRIPT Reverse Transcription Supermix (Bio-Rad). RT-PCR was performed using SYBR green on the CFX system (Bio-Rad). All assays were performed in triplicate. The averaged cycle of threshold (Ct) value of the two housekeeping genes (ACTIN/GAPDH) was subtracted from the Ct value of the gene of interest to obtain the ΔCt. Relative gene expression was determined as ^2−^ΔCt (ΔΔCt) and expressed relative to undifferentiated human embryonic stem cells (hESCs).

### Statistical analysis

Deviations from Hardy-Weinberg equilibrium were tested for using Pearson’s goodness-of-fit chi-square (degrees of freedom = 1), log likelihood ratio chi-square (degrees of freedom = 1), and Fisher’s Exact tests. De Finetti diagrams were also constructed [41]. Focusing on case-control (healthy individuals) phenotypes, we tested the null hypothesis of no association between rows and columns of the 2 × 3 matrix that contains the counts of the three genotypes (the two homozygotes and the heterozygote) among cases and controls. Genotype and allele frequencies were evaluated using Fisher’s exact test. We also performed the Armitage test (Monte Carlo method; it obtains results that are closer to those of an exact test, since the classical Cochran-Armitage trend test is based on approximation) [5]. All tests were performed as two-tailed, and differences were considered to be statistically significant when *P* < 0.05. Further calculations using contingency tables (chi-square test) were performed to explore data stratification, considering (i) the age of onset (below or above 50 years old), (ii) El Escorial criteria, and (iii) spinal or bulbar ALS onset.

## Results

### Whole-genome sequencing analysis of Greek sALS patients reveals a positive association with *FTO* and *TBC1D1* variants

Our next-generation sequencing-based genomics approach yielded a total of 174 SNPs that were found only in the sALS patients studied; these were variously intergenic (*n* = 144), intronic (*n* = 23), and/or were present in upstream (*n* = 2), downstream (*n* = 1), and 3′UTR (*n* = 2) regions or non-coding RNAs (*n* = 2). Next, genes were clustered by metabolic or disease network and the most prominent ones are shown in Table 3. Our data analysis revealed three genomic variants of prime interest: *FTO* rs2892469 (C>T) and rs1861869 (G>C) and *TBC1D1* rs6850200 (C>A). Findings were further explored in Greek sALS patients and their healthy counterparts (second patient cohort). *FTO* intronic variants, rs2892469 (C>T) and rs1861869 (G>C), reached statistical significance (*p* = 0.005 and *p* = 0.003, respectively). For *TBC1D1* rs6850200 (C>A), a strong association also became evident (*p* value of 0.017). Data are summarized in Tables 1 and 2 and Additional file 1.

**Table 3:** Gene clustering per metabolic or disease network using Ingenuity Pathway Analysis (IPA) to assist decision-making

Disease or metabolic network	Genes	*p* value
Neural cell proliferation	ISL1, FTO, ADCYAP1, ΟΤΡ, SPP1	6.02 × 10^–5^
Motor neuron proliferation	ISL1, FTO	9.53 × 10^–5^
Motor neuron dysfunction	ISL1, WNT4	1.28 × 10^–3^
Development of neuromuscular junction	PDZRN3	1.65 × 10^–3^
Branching of sensory neurons	PRKG1	1.65 × 10^–3^
ALS	FUS	1.65 × 10^–3^
Development of P and M cells of visual nervous system	ΟΤΡ	1.65 × 10^–3^
Growth of cerebellar cortex, regulation of upper cervical ganglia	ADCYAP1	1.65 × 10^–3^
Cranial nerve development	ISL1, ERBB4	1.9 × 10^–3^
Brain development	ADCYAP1, ERBB4, FTO, ΟΤΡ, PRKG1	2.56 × 10^–3^
Development of specific nerves of the hand, area of dendritic trees	ISL1	3.3 × 10^–3^
Movement of the cranial neural crest, participation in the formation of the central nervous system, astrocyte attachment	ERBB4	3.3 × 10^–3^
Neural cell number	ADCYAP1, ERBB4, WNT4, SPP1	3.75 × 10^–3^
Nervous system morphology	ERBB4, FTO, WNT4, SPP1, YES1, ISL1	6.33 × 10^–3^
Oligodendrocyte development, excitatory synapse formation	ERBB4	6.6 × 10^–3^
Central nervous system development, dendritic cell development	ERBB4, ADCYAP1	7.4 × 10^–3^
Motor neuron sprouting	FTO	9.88 × 10^–3^
v3 spinal cord motor neuron sprouting	FTO	9.88 × 10^–3^
Midbrain size	FTO	9.88 × 10^–3^
Neuronal withdrawal	WNT4	1.32 × 10^–2^
Brain morphology	ERBB4, FTO, SPP1, YES1	1.35 × 10^–2^
Neuronal branching	ERBB4, ADCYAP1, PRKG1	1.58 × 10^–2^
DNA repair	APTX, FTO, FUS	1.65 × 10^–2^

In addition, a *C9orf72* hexanucleotide expansion was identified in 1 of 28 patients with sALS (3.5%; Additional file 1: Table S4). This female patient was diagnosed with unequivocal ALS but was devoid of a positive family history for ALS.

### Linkage disequilibrium analyses suggest a haplotype of *FTO* gene variants

**Fig. 2:**
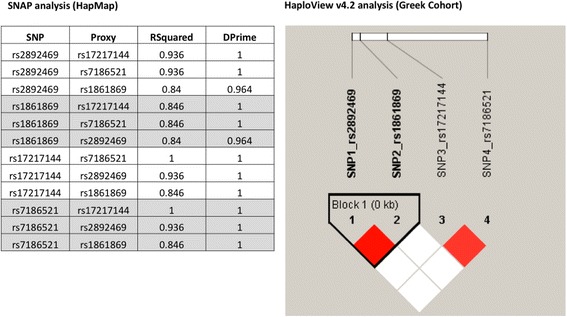
Linkage disequilibrium and haplotype block analysis for *FTO* rs2892469, rs1861869, rs17217144, and rs7186521. Data suggest a haplotype for the *FTO* genomic variants studied herein. SNAP analysis refers to HapMap datasets, while the HaploView v4.2 analysis corresponds to our Greek cohort. The HaploView 4.2 color scheme denotes the following: white (D′ < 1, LOD < 2) and red (D′ = 1, LOD ≥ 2). rs2892469 and rs1861869 are in perfect LD (D′ = 1, LOD = 23.66, *r* ^2^ = 0.884); rs17217144 and rs7186521 exhibit a strong LD (D′ = 0.882, LOD = 10.1, *r* ^2^ = 0.738). Block1 is based on confidence intervals, i.e., it was created through 95% of informative comparisons being in “strong LD”

Our findings support a disease-associated haplotype constituting *FTO* rs2892469 (C>T), rs1861869 (G>C), rs17217144 (C>T), and rs7186521 (A>G) (Fig. 2). SNAP analysis (HapMap datasets) was in agreement with the HaploView v4.2 analysis (our Greek cohort). The same result was produced for the LDmatrix module on the LDlink web tool. rs2892469 and rs1861869 are in perfect linkage disequilibrium (LD) (D′ = 1, LOD = 23.66, *r* ^2^ = 0.884), while rs17217144 and rs7186521 exhibit a strong LD (D′ = 0.882, LOD = 10.1, *r* ^2^ = 0.738). Block1 is based on confidence intervals, i.e., it was created due to 95% of informative comparisons being in “strong LD.” Such findings serve as a paradigm as to how information technology tools coupled to experimental data may empower discovery.

### In silico analyses suggest that *FTO* and *TBC1D1* variants affect splicing

*TBC1D1* rs6850200 (C>A) and *FTO* rs2892469 (C>T) were predicted to create a splicing enhancer site which would potentially alter splicing. In the case of *FTO* rs1861869 (G>C), a putative exonic splicing silencer site is disrupted, while *FTO* rs7186521 (A>G) appears to have created a new donor splice site. No impact on splicing was predicted for *FTO* rs17217144 (C>T). Using Variant Effect Predictor, PhyloP, and PhastCons data showed that the selected *FTO* variants are evolutionarily conserved.

### Replication analyses

Replication analyses were carried out in sALS patients of Greek origin (to ensure data reliability) and Sardinian and Turkish descent (to account for population differences (third patient cohort)). We found that four *FTO* intronic variants, rs2892469 (C>T) and rs1861869 (G>C), rs17217144 (C>T), and rs7186521 (A>G), reached statistical significance (*p* = 0.033, *p* = 0.07, *p* = 0.002, and *p* < 0.001, respectively), when Greek sALS patients were compared to their healthy counterparts. For *TBC1D1* rs6850200 (C>A), the aforementioned strong association (*p* value of 0.017) no longer held upon replication in Greek sALS patients (*p* value of 0.723). Data stratification based upon (i) the age of onset (below or above 50 years old), (ii) El Escorial criteria, and (iii) spinal or bulbar ALS onset did not alter our findings. It should be noted that all *FTO* and *TBC1D1* variants failed to reach statistical significance, even when stratified for *SOD1*, when Sardinian and Turkish sALS patients were considered. Data are summarized in Tables 1 and 2. The lack of association of these variants with sALS in patients of Sardinian and Turkish descent may suggest a founder effect in the Greek population.

### *FTO* is highly expressed in motor neurons

**Fig. 3:**
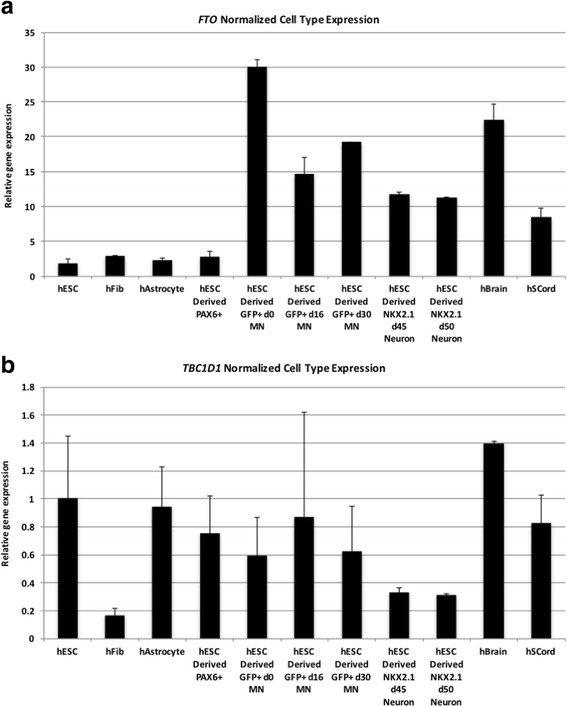
**a**, **b** *FTO* and *TBC1D1* expression levels in a range of various human cell types. *FTO* and *TBC1D1* mRNA levels were determined using human-specific primers in various cell types; fibroblasts, embryonic stem cells (ESCs), astrocytes, neural PAX6+ progenitors, stem cell-derived purified motor neurons at three different stages of development (Hb9:GFP on days 0, 16, and 30), and stem cell-derived cortical neurons (NKX21 on days 45 and 50). Human brain and spinal cord RNA served as controls. Normalized RT-qPCR data are expressed as averaged sample replicates. Error bars represent standard deviations

To begin defining genotype-to-phenotype associations for *FTO*, *TBC1D1*, and ALS, we determined the relative mRNA levels for both genes across several human cell types. We specifically analyzed fibroblasts, undifferentiated embryonic stem cells (ESCs), astrocytes, neural PAX6+ progenitors, and stem cell-derived spinal cord motor neurons (MNs) and cortical neurons (CNs). We used the HUES3-*Hb9*::GFP cell line, which contains a GFP reporter under the control of the MN-specific Hb9 enhancer [17] to differentiate and FACS-purify MNs as described previously [26], on three different stages of development (days 0, 16, and 30). We also used the *NKX2.1*::GFP ESC line to differentiate and FACS-purify cortical neurons as described previously [33] on three different stages of development (days 45 and 50). Our qRT-PCR data show that *FTO* is relatively neural specific and notably is most highly expressed in motor neurons (Fig. 3). *TBC1D1* seems to be rather ubiquitously expressed.

## Discussion

sALS is polygenic with a complex interplay with environmental and other factors. Previous studies have proposed genetic associations of a variety of genes with the etiology of sALS. In this article, we adopted a three-step approach to delineate the association of genomic variants with sALS in patients of Greek origin.

Using a next-generation sequencing-based genomics approach, we identified 174 variants that were only present in the initial sALS patients studied. We opted to focus on the *FTO* rs2892469 (C>T) and rs1861869 (G>C) and *TBC1D1* rs6850200 (C>A) variants, based on pathway analysis. Our replication analyses confirmed that the *FTO* but not the *TBC1D1* genomic variants maintained their statistical significance as far as their association with sALS was concerned. Data stratification considering (i) the age of onset (below or above 50 years old), (ii) El Escorial criteria, and (iii) spinal or bulbar ALS onset did not affect our findings (not shown).

We subsequently attempted to explore whether the *FTO* gene variants identified as being associated with sALS in Greek patients might also be associated with sALS in patients of Turkish and Sardinian descent. However, genetic analysis of all *FTO* and *TBC1D1* variants failed to reach statistical significance, even when stratified for *SOD1*, when Sardinian and Turkish sALS patients were considered. This may be due to a founder effect in the Greek population.

In order to explore the functional significance of our findings, we measured the *FTO* and *TBC1D1* gene expression levels in several human cell types, namely fibroblasts, embryonic stem cells (ESCs), astrocytes, neural PAX6+ progenitors, stem cell-derived purified motor neurons at three different stages of development (*Hb9*::GFP on days 0, 16, and 30), and stem cell-derived cortical neurons (NKX21 on days 45 and 50). Our qRT-PCR data demonstrated that *FTO* gene expression is relatively neuron-specific and notably is highly expressed in motor neurons (Fig. 3). By contrast, *TBC1D1* would appear to be rather ubiquitously expressed.

Interestingly, long distance interactions have been reported for the *FTO* intronic region: (i) obesity-associated non-coding sequences within *FTO* interact with the promoter regions of *IRX3* and *FTO* in human, mouse, and zebrafish, and (ii) obesity-associated *FTO* gene variants influence *IRX3* expression in the human brain. The latter gene was associated with body mass, as *IRX3*-deficient mice displayed a decrease in their body weight by 25–30% [42]. Nutritional surveillance of ALS patients has been well established to be of fundamental importance, both in bulbar and spinal onset patients, and nutritional status is a prognostic factor for survival in ALS patients [16]. Jawaid et al. [22] reported that a decrease in body mass index is associated with faster progression of motor symptoms and shorter survival in ALS patients. Moreover, an LD block of 49 kb in the first intron of *FTO* contains numerous highly conserved non-coding elements (HCNEs) and lies within an extended region of conserved synteny involving the *IRX3/5/6* cluster, the closest gene of which is the transcription factor-encoding *IRX3* [37]. *IRX3* is a member of the Iroquois homeobox gene family and plays a role in the early stages of neural development [7].

We also identified an example of *C9orf72* hexanucleotide expansion in 1 of 28 patients with sALS (3.5%; Additional file 1: Table S4). This female patient, harboring this expansion, was diagnosed with unequivocal ALS but was lacked a positive family history of ALS. This massive hexanucleotide repeat expansion mutation in *C9orf72*, which has been linked to fALS, sALS, and frontotemporal dementia, has a single founder who is thought to have originated approximately 6300 years ago in Europe [31, 43]. Our findings differ somewhat from those reported by Mok and coworkers [35], the only study to date in fALS and sALS patients of Greek descent, in which 11 of 136 sALS patients (8.2%) were carriers of the expansion. Such a discrepancy may reflect inter-individual differences not as far as statistical power is concerned, but rather related to the patients’ genetic background, as the aforementioned study examined only individuals from Athens, whereas we recruited individuals from across the entire Greek mainland (north, central, and south Greece). Differential exposure to environmental factors are known to contribute to an increased risk of ALS; thus, exposure to lead, mercury, selenium, zinc, and copper is known to increase the risk of ALS [46], while high-risk populations include those who collect their domestic water from surface water [8], possibly reflecting an association between the neurotoxin b-methylamino-l-alanine (BMAA) produced by cyanobacteria and the risk of ALS. Al-Chalabi and Hardiman [4] have proposed a disease model, according to which ALS is the net result of environmental risks and time acting on a pre-existing genetic load, followed by an automatic, self-perpetuating decline until death. Akimoto et al. [2] reported marked differences in genotyping results for GGGGCC-repeat expansions in *C9orf72.* Herein, amplicon length analysis and repeat-primed PCR were combined as a recommended minimum requirement in a research setting [2].

Small sample sizes and data variability, plus our ancestrally diverse patient cohorts, should be taken into account when interpreting our findings. Yet, our datasets may assist future efforts of replicating these findings in independent ALS patient cohorts.

## Conclusions

There is no single predominant genetic association underlying sALS. Instead, sALS may be considered to be the net result of the complex interplay of environmental factors and numerous low-risk susceptibility loci. We propose that under this polygenic model, a large number of alleles, each conferring a small genotypic risk, combine additively or multiplicatively to confer a range of susceptibilities in the general population [21, 47].

To our knowledge, this has been the first study to present a possible association between *FTO* variants and the genetic basis of sALS. These findings need to be further validated in a larger sALS patient cohort of Greek origin to overcome the small sample size bias, while a gene ontology analysis to look at links between *FTO* and the other genes known to be associated with ALS could support the validity of these findings.

## Additional file

Additional file 1: Supplementary Tables S1–3. (PDF 784 kb)

## Abbreviations

ALS: Amyotrophic lateral sclerosis
ESCs: Embryonic stem cells
fALS: Familial amyotrophic lateral sclerosis
HCNEs: Highly conserved non-coding elements
sALS: Sporadic amyotrophic lateral sclerosis
SNPs: Single nucleotide polymorphisms
