# Supplementary material for: Genomic variants in the FTO gene are associated with sporadic amyotrophic lateral sclerosis in Greek patients
Source: Hum Genomics. 2017 Dec 8;11:30. doi: 10.1186/s40246-017-0126-2 (PMC5721583; doi:10.1186/s40246-017-0126-2)
Supplement: Additional file 1: — Supplementary Tables S1–3. (PDF 784 kb) [file 40246_2017_126_MOESM1_ESM.pdf]

Supplementary Information

Genomic variants in the *FTO* gene are associated with sporadic amyotrophic lateral sclerosis in Greek patients

Konstantinos Mitropoulos[1,#], Eleni Merkouri Papadima[2,#,%], Georgia Xiromerisiou[3], Angeliki Balasopoulou[2], Kyriaki Charalampidou[2], Vasiliki Galani[2], Krystallia-Vassiliki Zafeiri[2], Efthymios Dardiotis[3], Styliani Ralli[3], Georgia Deretzi[4], Anne John[5], Kyriaki Kydonopoulou[6], Elpida Papadopoulou[6], Alba di Pardo[7], Fulya Akcimen[8], Annalisa Loizedda[9,10], Valerija Dobričić[11], Ivana Novaković[11,12], Vladimir S. Kostić[11], Clint Mizzi[2], Brock A. Peters[13,14], A. Nazli Basak[8], Sandro Orrù [9], Evangelos Kiskinis[7], David N. Cooper[15], Spyridon Gerou[6], Radoje Drmanac[13], Marina Bartsakoulia[2], Evangelia-Eirini Tsermpini[2], George M. Hadjigeorgiou[3], Bassam R. Ali[5], Theodora Katsila[2], George P. Patrinos[2,5,]*

[1] National and Kapodistrian University of Athens School of Medicine, Athens, Greece;

[2] Department of Pharmacy, University of Patras School of Health Sciences, Patras, Greece;

[3] School of Medicine, University of Thessaly, Larisa, Greece;

[4] Papageorgiou hospital, Thessaloniki, Greece;

[5] United Arab Emirates University, College of Medicine and Health Sciences, Department of Pathology, Al-Ain, UAE;

[6] ANALYSI Diagnostic Laboratories S.A., Thessaloniki, Greece;

[7] Departments of Neurology and Physiology, Northwestern University Feinberg School of Medicine, Chicago, IL, USA;

[8] Bogazici University, Suna and Inan Kirac Foundation, NDAL, Istanbul, Turkey;

[9] University of Cagliari, Department of Medical Sciences and Public Health, Cagliari, Italy;

[10] CNR IRGB, Cagliari, Italy;

[11] Institute of Neurology CCS, School of Medicine, University of Belgrade, Belgrade, Serbia;

[12] Faculty of Medicine, University of Belgrade, Institute of Human Genetics, Belgrade, Serbia;

[13] Complete Genomics Inc, Mountain View, CA, USA;

[14] BGI Shenzhen, Shenzhen, People's Republic of China;

[15] Cardiff University, Institute of Medical Genetics, Cardiff, UK

[#] These authors contributed equally to this work

[%] Present address: University of Cagliari, School of Medicine, Department of Biomedical Sciences, Cagliari, Italy

Supplementary Table 1. Statistical and Hardy-Weinberg analyses on the genotyping data of sALS patients and healthy individuals of Greek origin.

SNP	Tests for deviation from Hardy-Weinberg equilibrium		Tests for association (C.I.: 95% convidence interval)					
	Controls	Cases		allele freq. difference	heterozygous	homozygous	allele positivity	Armitage's trend test
rs17217144	n11=4 (17.54)<br>n12=59 (31.93)<br>n22=1 (14.54)<br>f_a1=0.52 +/-0.017<br>F=-0.84781<br>p=1.181e-11 (Pearson)<br>p=1.048e-13 (Llr)<br>p=1.711e-12 (Exact)	n11=30 (48.60)<br>n12=102 (64.80)<br>n22=3 (21.60)<br>f_a1=0.60 +/-0.019<br>F=-0.57407<br>p=2.556e-11 (Pearson)<br>p=6.278e-13 (Llr)<br>p=3.652e-12 (Exact)	Risk allele 2	[1]<->[2]<br>Odds_ratio=0.732<br>C.I.=[0.479-1.119]<br>chi2=2.08<br>p=0.14891 (P)	[11]<->[12]<br>Odds_ratio=0.231<br>C.I.=[0.077-0.687]<br>chi2=7.95<br>p=0.00482	[11+]<->[22]<br>Odds_ratio=0.400<br>C.I.=[0.033-4.834]<br>chi2=0.55<br>p=0.45886	[11]<->[12+22]<br>Odds_ratio=0.233<br>C.I.=[0.078-0.694]<br>chi2=7.82<br>p=0.00517	common odds ratio<br>Odds_ratio=0.379<br>chi2=6.05<br>p=0.01390
			Risk allele 1	[2]<->[1]<br>Odds_ratio=1.366<br>C.I.=[0.894-2.086]<br>chi2=2.08<br>p=0.14891 (P)	[22]<->[12]<br>Odds_ratio=0.576<br>C.I.=[0.059-5.667]<br>chi2=0.23<br>p=0.63244	[22]<->[11]<br>Odds_ratio=2.500<br>C.I.=[0.207-30.215]<br>chi2=0.55<br>p=0.45886	[11+12]<->[22]<br>Odds_ratio=0.698<br>C.I.=[0.071-6.849]<br>chi2=0.10<br>p=0.75676	common odds ratio<br>Odds_ratio=2.490<br>chi2=6.05<br>p=0.01390
rs2892469	n11=10 (7.85)<br>n12=15 (19.29)<br>n22=14 (11.85)<br>f_a1=0.45 +/-0.062<br>F=0.22259<br>p=0.164504 (Pearson)<br>p=0.163279 (Llr)<br>p=0.196593 (Exact)	n11=30 (34.26)<br>n12=79 (70.49)<br>n22=32 (36.26)<br>f_a1=0.49 +/-0.028<br>F=-0.12079<br>p=0.151476 (Pearson)<br>p=0.150969 (Llr)<br>p=0.180121 (Exact)	Risk allele 2	[1]<->[2]<br>Odds_ratio=0.837<br>C.I.=[0.506-1.386]<br>chi2=0.48<br>p=0.48943 (P)	[11]<->[12]<br>Odds_ratio=1.756<br>C.I.=[0.711-4.335]<br>chi2=1.51<br>p=0.21886	[11+]<->[22]<br>Odds_ratio=0.762<br>C.I.=[0.294-1.974]<br>chi2=0.31<br>p=0.57517	[11]<->[12+22]<br>Odds_ratio=1.276<br>C.I.=[0.560-2.909]<br>chi2=0.34<br>p=0.56175	common odds ratio<br>Odds_ratio=0.855<br>chi2=0.50<br>p=0.47923
			Risk allele 1	[2]<->[1]<br>Odds_ratio=1.194<br>C.I.=[0.722-1.976]<br>chi2=0.48<br>p=0.48943 (P)	[22]<->[12]<br>Odds_ratio=2.304<br>C.I.=[0.999-5.317]<br>chi2=3.94<br>p=0.04711	[22]<->[11]<br>Odds_ratio=1.312<br>C.I.=[0.506-3.401]<br>chi2=0.31<br>p=0.57517	[11+12]<->[22]<br>Odds_ratio=1.907<br>C.I.=[0.889-4.094]<br>chi2=2.80<br>p=0.09432	common odds ratio<br>Odds_ratio=1.167<br>chi2=0.50<br>p=0.47923
rs7186521	n11=0 (12.25)<br>n12=49 (24.50)<br>n22=0 (12.25)<br>f_a1=0.50 +/-0.000<br>F=-1.00000<br>p=2.560e-12 (Pearson)<br>p=1.695e-16 (Llr)<br>p=2.210e-14 (Exact)	n11=24 (9.46)<br>n12=24 (53.08)<br>n22=89 (74.46)<br>f_a1=0.26 +/-0.033<br>F=0.54785<br>p=1.432e-10 (Pearson)<br>p=5.947e-10 (Llr)<br>p=7.520e-10 (Exact)	Risk allele 2	[1]<->[2]<br>Odds_ratio=2.806<br>C.I.=[1.738-4.528]<br>chi2=18.51<br>p=0.00002 (P)	[11]<->[12]<br>Odds_ratio=0.010<br>C.I.=[0.001-0.173]<br>chi2=32.55<br>p=1.159e-08	[11+]<->[22]<br>Odds_ratio=3.653<br>C.I.=[0.071-188.837]<br>chi2=nan<br>p=1.00000	[11]<->[12+22]<br>Odds_ratio=0.047<br>C.I.=[0.003-0.785]<br>chi2=9.86<br>p=0.00169	common odds ratio<br>Odds_ratio=2.184<br>chi2=16.74<br>p=0.00004
			Risk allele 1	[2]<->[1]<br>Odds_ratio=0.356<br>C.I.=[0.221-0.575]<br>chi2=18.51<br>p=0.00002 (P)	[22]<->[12]<br>Odds_ratio=0.003<br>C.I.=[0.000-0.046]<br>chi2=85.64<br>p=2.154e-20	[22]<->[11]<br>Odds_ratio=0.274<br>C.I.=[0.005-14.151]<br>chi2=nan<br>p=1.00000	[11+12]<->[22]<br>Odds_ratio=0.005<br>C.I.=[0.000-0.091]<br>chi2=61.04<br>p=5.596e-15	common odds ratio<br>Odds_ratio=0.456<br>chi2=16.74<br>p=0.00004
rs1861869	n11=8 (6.16)<br>n12=15 (18.68)<br>n22=16 (14.16)<br>f_a1=0.40 +/-0.061<br>F=0.19698<br>p=0.218645 (Pearson)<br>p=0.219340 (Llr)<br>p=0.312376 (Exact)	n11=24 (27.46)<br>n12=76 (69.09)<br>n22=40 (43.46)<br>f_a1=0.44 +/-0.028<br>F=-0.10008<br>p=0.236336 (Pearson)<br>p=0.235483 (Llr)<br>p=0.304045 (Exact)	Risk allele 2	[1]<->[2]<br>Odds_ratio=0.830<br>C.I.=[0.498-1.383]<br>chi2=0.51<br>p=0.47399 (P)	[11]<->[12]<br>Odds_ratio=1.689<br>C.I.=[0.638-4.469]<br>chi2=1.13<br>p=0.28788	[11+]<->[22]<br>Odds_ratio=0.833<br>C.I.=[0.310-2.238]<br>chi2=0.13<br>p=0.71745	[11]<->[12+22]<br>Odds_ratio=1.247<br>C.I.=[0.511-3.046]<br>chi2=0.24<br>p=0.62714	common odds ratio<br>Odds_ratio=0.869<br>chi2=0.53<br>p=0.46600
			Risk allele 1	[2]<->[1]<br>Odds_ratio=1.205<br>C.I.=[0.723-2.009]<br>chi2=0.51<br>p=0.47399 (P)	[22]<->[12]<br>Odds_ratio=2.027<br>C.I.=[0.909-4.518]<br>chi2=3.04<br>p=0.08104	[22]<->[11]<br>Odds_ratio=1.200<br>C.I.=[0.447-3.223]<br>chi2=0.13<br>p=0.71745	[11+12]<->[22]<br>Odds_ratio=1.739<br>C.I.=[0.833-3.630]<br>chi2=2.20<br>p=0.13794	common odds ratio<br>Odds_ratio=1.148<br>chi2=0.53<br>p=0.46600
rs6850200	n11=9 (8.04)<br>n12=27 (28.93)<br>n22=27 (26.04)<br>f_a1=0.36 +/-0.044<br>F=0.06667<br>p=0.596701 (Pearson)<br>p=0.597914 (Llr)<br>p=0.589957 (Exact)	n11=15 (16.68)<br>n12=67 (63.63)<br>n22=59 (60.68)<br>f_a1=0.34 +/-0.028<br>F=-0.05288<br>p=0.530030 (Pearson)<br>p=0.528265 (Llr)<br>p=0.580852 (Exact)	Risk allele 2	[1]<->[2]<br>Odds_ratio=1.060<br>C.I.=[0.683-1.644]<br>chi2=0.07<br>p=0.79638 (P)	[11]<->[12]<br>Odds_ratio=1.489<br>C.I.=[0.582-3.809]<br>chi2=0.69<br>p=0.40459	[11+]<->[22]<br>Odds_ratio=1.311<br>C.I.=[0.510-3.368]<br>chi2=0.32<br>p=0.57305	[11]<->[12+22]<br>Odds_ratio=1.400<br>C.I.=[0.577-3.395]<br>chi2=0.56<br>p=0.45505	common odds ratio<br>Odds_ratio=1.096<br>chi2=0.07<br>p=0.79483
			Risk allele 1	[2]<->[1]<br>Odds_ratio=0.944<br>C.I.=[0.608-1.465]<br>chi2=0.07<br>p=0.79638 (P)	[22]<->[12]<br>Odds_ratio=1.136<br>C.I.=[0.600-2.149]<br>chi2=0.15<br>p=0.69598	[22]<->[11]<br>Odds_ratio=0.763<br>C.I.=[0.297-1.959]<br>chi2=0.32<br>p=0.57305	[11+12]<->[22]<br>Odds_ratio=1.042<br>C.I.=[0.572-1.901]<br>chi2=0.02<br>p=0.89230	common odds ratio<br>Odds_ratio=0.915<br>chi2=0.07<br>p=0.79483

(Pearson): Pearson's goodness-of-fit chi-square (degrees of freedom=1); (Llr): Log likelihood ratio chi-square (degrees of freedom=1); (Exact): Fisher's Exact test PA, PB: two-tailed p-values; if P, then PA=PB

Supplementary Table 2. Statistical and Hardy-Weinberg analyses on the genotyping data of sALS patients and healthy individuals of Sardinian origin.

SNP	Tests for deviation from Hardy-Weinberg equilibrium		Tests for association (C.I.: 95% convidence interval)				
	Controls	Cases	allele freq. difference	heterozygous	homozygous	allele positivity	Armitage's trend test
rs6850200	n11=17 (13.29)<br>n12=34 (41.43)<br>n22=36 (32.29)<br>f_a1=0.39 +/-0.040<br>F=0.17925<br>p=0.094546 (Pearson)<br>p=0.095316 (Llr)<br>p=0.113989 (Exact)	n11=17 (16.22)<br>n12=52 (53.56)<br>n22=45 (44.22)<br>f_a1=0.38 +/-0.033<br>F=0.02915<br>p=0.755607 (Pearson)<br>p=0.755876 (Llr)<br>p=0.841794 (Exact)	Risk allele 2				
			[1]<->[2]	[11]<->[12]	[11+]<->[22]	[11]<->[12+22]	common odds ratio
			Odds_ratio=1.059<br>C.I.=[0.706-1.589]<br>chi2=0.08<br>p=0.78090 (P)	Odds_ratio=1.529<br>C.I.=[0.688-3.401]<br>chi2=1.09<br>p=0.29603	Odds_ratio=1.250<br>C.I.=[0.560-2.789]<br>chi2=0.30<br>p=0.58547	Odds_ratio=1.386<br>C.I.=[0.662-2.902]<br>chi2=0.75<br>p=0.38585	Odds_ratio=1.083<br>chi2=0.07<br>p=0.79037
			Risk allele 1				
			[2]<->[1]	[22]<->[12]	[22]<->[11]	[11+12]<->[22]	common odds ratio
			Odds_ratio=0.944<br>C.I.=[0.629-1.416]<br>chi2=0.08<br>p=0.78090 (P)	Odds_ratio=1.224<br>C.I.=[0.661-2.264]<br>chi2=0.41<br>p=0.52047	Odds_ratio=0.800<br>C.I.=[0.359-1.785]<br>chi2=0.30<br>p=0.58547	Odds_ratio=1.082<br>C.I.=[0.613-1.911]<br>chi2=0.07<br>p=0.78492	Odds_ratio=0.925<br>chi2=0.07<br>p=0.79037
rs2892469	n11=32 (27.11)<br>n12=32 (41.79)<br>n22=21 (16.11)<br>f_a1=0.56 +/-0.042<br>F=0.23423<br>p=0.030809 (Pearson)<br>p=0.030392 (Llr)<br>p=0.029812 (Exact)	n11=32 (27.27)<br>n12=42 (51.46)<br>n22=29 (24.27)<br>f_a1=0.51 +/-0.038<br>F=0.18377<br>p=0.062168 (Pearson)<br>p=0.061449 (Llr)<br>p=0.075147 (Exact)	Risk allele 2				
			[1]<->[2]	[11]<->[12]	[11+]<->[22]	[11]<->[12+22]	common odds ratio
			Odds_ratio=1.224<br>C.I.=[0.814-1.841]<br>chi2=0.94<br>p=0.33178 (P)	Odds_ratio=1.312<br>C.I.=[0.670-2.570]<br>chi2=0.63<br>p=0.42735	Odds_ratio=1.381<br>C.I.=[0.655-2.910]<br>chi2=0.72<br>p=0.39544	Odds_ratio=1.340<br>C.I.=[0.731-2.455]<br>chi2=0.90<br>p=0.34339	Odds_ratio=1.179<br>chi2=0.78<br>p=0.37729
			Risk allele 1				
			[2]<->[1]	[22]<->[12]	[22]<->[11]	[11+12]<->[22]	common odds ratio
			Odds_ratio=0.817<br>C.I.=[0.543-1.229]<br>chi2=0.94<br>p=0.33178 (P)	Odds_ratio=0.950<br>C.I.=[0.460-1.964]<br>chi2=0.02<br>p=0.89081	Odds_ratio=0.724<br>C.I.=[0.344-1.526]<br>chi2=0.72<br>p=0.39544	Odds_ratio=0.837<br>C.I.=[0.435-1.610]<br>chi2=0.28<br>p=0.59420	Odds_ratio=0.848<br>chi2=0.78<br>p=0.37729
rs1861869	n11=31 (23.82)<br>n12=28 (42.35)<br>n22=26 (18.82)<br>f_a1=0.53 +/-0.044<br>F=0.33889<br>p=0.001782 (Pearson)<br>p=0.001612 (Llr)<br>p=0.002133 (Exact)	n11=30 (23.31)<br>n12=38 (51.38)<br>n22=35 (28.31)<br>f_a1=0.48 +/-0.039<br>F=0.26039<br>p=0.008225 (Pearson)<br>p=0.007881 (Llr)<br>p=0.009902 (Exact)	Risk allele 2				
			[1]<->[2]	[11]<->[12]	[11+]<->[22]	[11]<->[12+22]	common odds ratio
			Odds_ratio=1.240<br>C.I.=[0.825-1.862]<br>chi2=1.07<br>p=0.30012 (P)	Odds_ratio=1.402<br>C.I.=[0.696-2.825]<br>chi2=0.90<br>p=0.34325	Odds_ratio=1.391<br>C.I.=[0.681-2.840]<br>chi2=0.82<br>p=0.36424	Odds_ratio=1.397<br>C.I.=[0.757-2.579]<br>chi2=1.15<br>p=0.28439	Odds_ratio=1.180<br>chi2=0.83<br>p=0.36307
			Risk allele 1				
			[2]<->[1]	[22]<->[12]	[22]<->[11]	[11+12]<->[22]	common odds ratio
			Odds_ratio=0.807<br>C.I.=[0.537-1.211]<br>chi2=1.07<br>p=0.30012 (P)	Odds_ratio=1.008<br>C.I.=[0.499-2.039]<br>chi2=0.00<br>p=0.98194	Odds_ratio=0.719<br>C.I.=[0.352-1.468]<br>chi2=0.82<br>p=0.36424	Odds_ratio=0.856<br>C.I.=[0.463-1.585]<br>chi2=0.24<br>p=0.62097	Odds_ratio=0.848<br>chi2=0.83<br>p=0.36307
rs17217144	n11=20 (16.11)<br>n12=34 (41.79)<br>n22=31 (27.11)<br>f_a1=0.44 +/-0.041<br>F=0.18637<br>p=0.085745 (Pearson)<br>p=0.085386 (Llr)<br>p=0.082697 (Exact)	n11=28 (24.27)<br>n12=44 (51.46)<br>n22=31 (27.27)<br>f_a1=0.49 +/-0.037<br>F=0.14491<br>p=0.141391 (Pearson)<br>p=0.140719 (Llr)<br>p=0.166760 (Exact)	Risk allele 2				
			[1]<->[2]	[11]<->[12]	[11+]<->[22]	[11]<->[12+22]	common odds ratio
			Odds_ratio=0.817<br>C.I.=[0.543-1.229]<br>chi2=0.94<br>p=0.33178 (P)	Odds_ratio=0.924<br>C.I.=[0.446-1.914]<br>chi2=0.04<br>p=0.83224	Odds_ratio=0.714<br>C.I.=[0.334-1.527]<br>chi2=0.76<br>p=0.38474	Odds_ratio=0.824<br>C.I.=[0.425-1.599]<br>chi2=0.33<br>p=0.56730	Odds_ratio=0.843<br>chi2=0.81<br>p=0.36868
			Risk allele 1				
			[2]<->[1]	[22]<->[12]	[22]<->[11]	[11+12]<->[22]	common odds ratio
			Odds_ratio=1.224<br>C.I.=[0.814-1.841]<br>chi2=0.94<br>p=0.33178 (P)	Odds_ratio=1.294<br>C.I.=[0.663-2.528]<br>chi2=0.57<br>p=0.44999	Odds_ratio=1.400<br>C.I.=[0.655-2.993]<br>chi2=0.76<br>p=0.38474	Odds_ratio=1.333<br>C.I.=[0.724-2.454]<br>chi2=0.86<br>p=0.35490	Odds_ratio=1.186<br>chi2=0.81<br>p=0.36868
rs7186521	n11=30 (27.11)<br>n12=36 (41.79)<br>n22=19 (16.11)<br>f_a1=0.56 +/-0.041<br>F=0.13851<br>p=0.201591 (Pearson)<br>p=0.201488 (Llr)<br>p=0.193664 (Exact)	n11=30 (27.79)<br>n12=47 (51.42)<br>n22=26 (23.79)<br>f_a1=0.52 +/-0.036<br>F=0.08600<br>p=0.382769 (Pearson)<br>p=0.382512 (Llr)<br>p=0.429829 (Exact)	Risk allele 2				
			[1]<->[2]	[11]<->[12]	[11+]<->[22]	[11]<->[12+22]	common odds ratio
			Odds_ratio=1.200<br>C.I.=[0.798-1.805]<br>chi2=0.77<br>p=0.38052 (P)	Odds_ratio=1.306<br>C.I.=[0.670-2.543]<br>chi2=0.62<br>p=0.43279	Odds_ratio=1.368<br>C.I.=[0.628-2.981]<br>chi2=0.62<br>p=0.42920	Odds_ratio=1.327<br>C.I.=[0.717-2.456]<br>chi2=0.82<br>p=0.36655	Odds_ratio=1.174<br>chi2=0.69<br>p=0.40550
			Risk allele 1				
			[2]<->[1]	[22]<->[12]	[22]<->[11]	[11+12]<->[22]	common odds ratio
			Odds_ratio=0.833<br>C.I.=[0.554-1.253]<br>chi2=0.77<br>p=0.38052 (P)	Odds_ratio=0.954<br>C.I.=[0.458-1.987]<br>chi2=0.02<br>p=0.90003	Odds_ratio=0.731<br>C.I.=[0.335-1.592]<br>chi2=0.62<br>p=0.42920	Odds_ratio=0.853<br>C.I.=[0.433-1.677]<br>chi2=0.21<br>p=0.64396	Odds_ratio=0.851<br>chi2=0.69<br>p=0.40550

(Pearson): Pearson's goodness-of-fit chi-square (degrees of freedom=1); (Llr): Log likelihood ratio chi-square (degrees of freedom=1); (Exact): Fisher’s Exact test PA, PB: two-tailed p-values; if P, then PA=PB

Supplementary Table 3. Statistical and Hardy-Weinberg analyses on the genotyping data of sALS patients and healthy individuals of Turkish origin.

SNP	Tests for deviation from Hardy-Weinberg equilibrium		Tests for association (C.I.: 95% convidence interval)				
	Controls	Cases	allele freq. difference	heterozygous	homozygous	allele positivity	Armitage's trend test
rs6850200	n11=13 (9.85)<br>n12=28 (34.30)<br>n22=33 (29.85)<br>f_a1=0.36 +/-0.043<br>F=0.18361<br>p=0.114229 (Pearson)<br>p=0.116119 (Llr)<br>p=0.132161 (Exact)	n11=19 (17.57)<br>n12=64 (66.85)<br>n22=65 (63.57)<br>f_a1=0.34 +/-0.028<br>F=0.04265<br>p=0.603841 (Pearson)<br>p=0.604834 (Llr)<br>p=0.588753 (Exact)	**Risk allele 2**				
			**[1]<->[2]**	**[11]<->[12]**	**[11+]<->[22]**	**[11]<->[12+22]**	**common odds ratio**
			Odds_ratio=1.093<br>C.I.=[0.724-1.649]<br>chi2=0.18<br>p=0.67320 (P)	Odds_ratio=1.564<br>C.I.=[0.679-3.599]<br>chi2=1.11<br>p=0.29123	Odds_ratio=1.348<br>C.I.=[0.593-3.061]<br>chi2=0.51<br>p=0.47520	Odds_ratio=1.447<br>C.I.=[0.671-3.120]<br>chi2=0.89<br>p=0.34424	Odds_ratio=1.119<br>chi2=0.16<br>p=0.68634
			**Risk allele 1**				
			**[2]<->[1]**	**[22]<->[12]**	**[22]<->[11]**	**[11+12]<->[22]**	**common odds ratio**
			Odds_ratio=0.915<br>C.I.=[0.606-1.381]<br>chi2=0.18<br>p=0.67320 (P)	Odds_ratio=1.160<br>C.I.=[0.630-2.137]<br>chi2=0.23<br>p=0.63276	Odds_ratio=0.742<br>C.I.=[0.327-1.686]<br>chi2=0.51<br>p=0.47520	Odds_ratio=1.028<br>C.I.=[0.586-1.802]<br>chi2=0.01<br>p=0.92386	Odds_ratio=0.897<br>chi2=0.16<br>p=0.68634
rs2892469	n11=19 (18.50)<br>n12=36 (37.00)<br>n22=19 (18.50)<br>f_a1=0.50 +/-0.042<br>F=0.02703<br>p=0.816153 (Pearson)<br>p=0.816142 (Llr)<br>p=0.818460 (Exact)	n11=36 (39.54)<br>n12=81 (73.92)<br>n22=31 (34.54)<br>f_a1=0.52 +/-0.028<br>F=-0.09585<br>p=0.243611 (Pearson)<br>p=0.243211 (Llr)<br>p=0.322762 (Exact)	**Risk allele 2**				
			**[1]<->[2]**	**[11]<->[12]**	**[11+]<->[22]**	**[11]<->[12+22]**	**common odds ratio**
			Odds_ratio=0.935<br>C.I.=[0.630-1.387]<br>chi2=0.11<br>p=0.73712 (P)	Odds_ratio=1.188<br>C.I.=[0.601-2.345]<br>chi2=0.25<br>p=0.62043	Odds_ratio=0.861<br>C.I.=[0.388-1.910]<br>chi2=0.14<br>p=0.71295	Odds_ratio=1.075<br>C.I.=[0.565-2.044]<br>chi2=0.05<br>p=0.82598	Odds_ratio=0.930<br>chi2=0.12<br>p=0.72993
			**Risk allele 1**				
			**[2]<->[1]**	**[22]<->[12]**	**[22]<->[11]**	**[11+12]<->[22]**	**common odds ratio**
			Odds_ratio=1.070<br>C.I.=[0.721-1.588]<br>chi2=0.11<br>p=0.73712 (P)	Odds_ratio=1.379<br>C.I.=[0.690-2.758]<br>chi2=0.83<br>p=0.36250	Odds_ratio=1.161<br>C.I.=[0.523-2.576]<br>chi2=0.14<br>p=0.71295	Odds_ratio=1.304<br>C.I.=[0.677-2.510]<br>chi2=0.63<br>p=0.42646	Odds_ratio=1.075<br>chi2=0.12<br>p=0.72993
rs1861869	n11=0 (0.00)<br>n12=0 (0.00)<br>n22=74 (0.00)<br>f_a1=0.00 +/-0.000<br>F=0.00000<br>p=0.000e+00 (Pearson)<br>p=0.000e+00 (Llr)<br>p=0.000e+00 (Exact)	n11=0 (0.00)<br>n12=1 (1.00)<br>n22=137 (137.00)<br>f_a1=0.00 +/-0.004<br>F=-0.00364<br>p=0.965927 (Pearson)<br>p=0.951915 (Llr)<br>p=1.000000 (Exact)	**Risk allele 2**				
			**[1]<->[2]**	**[11]<->[12]**	**[11+]<->[22]**	**[11]<->[12+22]**	**common odds ratio**
			Odds_ratio=0.618<br>C.I.=[0.025-15.275]<br>chi2=0.54<br>p=1.11118 (F)	Odds_ratio=3.000<br>C.I.=[0.019-473.058]<br>chi2=nan<br>p=1.00000	Odds_ratio=1.846<br>C.I.=[0.036-93.961]<br>chi2=nan<br>p=1.00000	Odds_ratio=1.859<br>C.I.=[0.037-94.642]<br>chi2=nan<br>p=1.00000	Odds_ratio=1.197<br>chi2=0.54<br>p=0.46294
			**Risk allele 1**				
			**[2]<->[1]**	**[22]<->[12]**	**[22]<->[11]**	**[11+12]<->[22]**	**common odds ratio**
			Odds_ratio=1.617<br>C.I.=[0.065-39.942]<br>chi2=0.54<br>p=1.11118 (F)	Odds_ratio=1.625<br>C.I.=[0.065-40.398]<br>chi2=0.54<br>p=0.46294	Odds_ratio=0.542<br>C.I.=[0.011-27.584]<br>chi2=nan<br>p=1.00000	Odds_ratio=1.625<br>C.I.=[0.065-40.398]<br>chi2=0.54<br>p=0.46294	Odds_ratio=0.858<br>chi2=0.54<br>p=0.46294
rs17217144	n11=19 (18.50)<br>n12=36 (37.00)<br>n22=19 (18.50)<br>f_a1=0.50 +/-0.042<br>F=0.02703<br>p=0.816153 (Pearson)<br>p=0.816142 (Llr)<br>p=0.818460 (Exact)	n11=38 (41.11)<br>n12=80 (73.78)<br>n22=30 (33.11)<br>f_a1=0.53 +/-0.028<br>F=-0.08425<br>p=0.305394 (Pearson)<br>p=0.305020 (Llr)<br>p=0.409011 (Exact)	**Risk allele 2**				
			**[1]<->[2]**	**[11]<->[12]**	**[11+]<->[22]**	**[11]<->[12+22]**	**common odds ratio**
			Odds_ratio=0.897<br>C.I.=[0.605-1.332]<br>chi2=0.29<br>p=0.59108 (P)	Odds_ratio=1.111<br>C.I.=[0.565-2.186]<br>chi2=0.09<br>p=0.76022	Odds_ratio=0.789<br>C.I.=[0.356-1.750]<br>chi2=0.34<br>p=0.56021	Odds_ratio=1.000<br>C.I.=[0.528-1.894]<br>chi2=0.00<br>p=1.00000	Odds_ratio=0.891<br>chi2=0.30<br>p=0.58219
			**Risk allele 1**				
			**[2]<->[1]**	**[22]<->[12]**	**[22]<->[11]**	**[11+12]<->[22]**	**common odds ratio**
			Odds_ratio=1.114<br>C.I.=[0.751-1.654]<br>chi2=0.29<br>p=0.59108 (P)	Odds_ratio=1.407<br>C.I.=[0.701-2.824]<br>chi2=0.93<br>p=0.33514	Odds_ratio=1.267<br>C.I.=[0.571-2.808]<br>chi2=0.34<br>p=0.56021	Odds_ratio=1.359<br>C.I.=[0.704-2.623]<br>chi2=0.84<br>p=0.35996	Odds_ratio=1.122<br>chi2=0.30<br>p=0.58219
rs7186521	n11=19 (18.50)<br>n12=36 (37.00)<br>n22=19 (18.50)<br>f_a1=0.50 +/-0.042<br>F=0.02703<br>p=0.816153 (Pearson)<br>p=0.816142 (Llr)<br>p=0.818460 (Exact)	n11=31 (33.58)<br>n12=79 (73.83)<br>n22=38 (40.58)<br>f_a1=0.48 +/-0.028<br>F=-0.06996<br>p=0.394706 (Pearson)<br>p=0.394460 (Llr)<br>p=0.509316 (Exact)	**Risk allele 2**				
			**[1]<->[2]**	**[11]<->[12]**	**[11+]<->[22]**	**[11]<->[12+22]**	**common odds ratio**
			Odds_ratio=1.099<br>C.I.=[0.741-1.631]<br>chi2=0.22<br>p=0.63832 (P)	Odds_ratio=1.345<br>C.I.=[0.672-2.692]<br>chi2=0.70<br>p=0.40176	Odds_ratio=1.226<br>C.I.=[0.554-2.710]<br>chi2=0.25<br>p=0.61477	Odds_ratio=1.304<br>C.I.=[0.677-2.510]<br>chi2=0.63<br>p=0.42646	Odds_ratio=1.105<br>chi2=0.23<br>p=0.63194
			**Risk allele 1**				
			**[2]<->[1]**	**[22]<->[12]**	**[22]<->[11]**	**[11+12]<->[22]**	**common odds ratio**
			Odds_ratio=0.910<br>C.I.=[0.613-1.350]<br>chi2=0.22<br>p=0.63832 (P)	Odds_ratio=1.097<br>C.I.=[0.557-2.160]<br>chi2=0.07<br>p=0.78826	Odds_ratio=0.816<br>C.I.=[0.369-1.804]<br>chi2=0.25<br>p=0.61477	Odds_ratio=1.000<br>C.I.=[0.528-1.894]<br>chi2=0.00<br>p=1.00000	Odds_ratio=0.905<br>chi2=0.23<br>p=0.63194

(Pearson): Pearson's goodness-of-fit chi-square (degrees of freedom=1); (Llr): Log likelihood ratio chi-square (degrees of freedom=1); (Exact): Fisher's Exact test PA, PB: two-tailed p-values; if P, then PA=PB

Supplementary Table 4. *C9orf72* genotyping of Greek ALS patients (second patient cohort)

Sample ID	*C9orf72* repeats
1	6/9
2	2/5
**3**	2/7
4	5/10
5	2/2
6	7/10
7	2/8
8	2/5
9	2/2
10	2/3
11	5/6
12	8/10
13	Inconclusive
14	2/2
15	2/8
16	2/5
17	2/2
18	2/2
19	2/2
20	2/5
21	2/8
22	2/5
23	2/2
24	2/4
25	Inconclusive
**26**	**2/exp**
27	2/7
28	2/2
29	6/11
30	6/11
31	No amplification

Numbers given in the table (column *C9orf72* results) represent the numbers of *C9orf72* hexanucleotide repeats for a given patient. For example, 2/6 means that the patient has 2 repeats on one allele, and 6 repeats on the other. 2/2 means that the patient in question is homozygous for allele 2. For patient 26, 2/exp means that there are 2 repeats on one allele and an expansion on the other. The size of the expansion cannot be defined because we use repeat-primed PCR.
